# Supplementary material for: Microphytoplankton variations during coral spawning at Los Roques, Southern Caribbean
Source: PeerJ. 2016 Mar 17;4:e1747. doi: 10.7717/peerj.1747 (PMC4806606; doi:10.7717/peerj.1747)
Supplement: Table S1 [file peerj-04-1747-s001.docx]

**Supplementary Table 1.** Microalgae species identified in water samples from four reefs, in nine consecutive days before, during and after coral spawning in 2007 and 2008 at Los Roques, Venezuela, Southern Caribbean.

| **Ochrophyta** |  | **Presence** | |
| --- | --- | --- | --- |
| **Bacillariophyceae** |  | **2007** | **2008** |
| *Actinoptychus* sp*.* | Shadboldt, G. (1854) | X | X |
| *Asterionellopsis glacialis* | (Castracane) Round, 1990 | X |  |
| *Aulacoseira* sp*.* | Thwaites, 1848 | X | X |
| *Cerataulina pelagica* | (Cleve) Hendey 1937 | X | X |
| *Chaetoceros* spp*.* | Ehrenberg, 1844 | X |  |
| *Chaetoceros decipiens* | Cleve, 1873 | X |  |
| *Chaetoceros peruvianus* | Brightwell, 1856 | X |  |
| *Chaetoceros socialis* | H.S.Lauder, 1864 | X |  |
| *Cyclostephanopsis* sp*.* | L. Loginova 1993 | X |  |
| *Cyclostephanos* sp*.* | Theriot et al. E.F. 1988 | X |  |
| *Climacosphenia* sp*.* | Ehrenberg, 1841 | X |  |
| *Closterium navicula* | (Brébisson) Lütkemüller, 1905 | X | X |
| *Corethron* sp*.* | Castracane, 1886 | X |  |
| *Cyclotella* sp*.* | (Kützing) Brébisson, 1838 | X |  |
| *Dactyliosolen* sp*.* | Castracane, [C.A.] F. 1886 | X | X |
| *Eucampia* sp*.* | Ehrenberg, 1839 | X | X |
| *Eucampia cornuta* | (Cleve) Grunow, 1883 | X | X |
| *Eunotia* sp*.* | Ehrenberg, 1837 | X |  |
| *Grammatophora* spp*.* | Ehrenberg, 1840 | X | X |
| *Hemiaulus hauckii* | Grunow ex Van Heurck, 1882 | X | X |
| *Hemiaulus membranaceus* | Cleve | X | X |
| *Lauderia* sp*.* | Cleve, 1873 | X |  |
| *Leptocylindrus danicus* | Cleve, 1889 | X | X |
| *Licmophora* spp*.* | C.Agardh, 1827 |  | X |
| *Lithodesmium* sp*.* | Ehrenberg, 1839 | X | X |
| *Lyrella* sp*.* | Karajeva, 1978 | X |  |
| *Mastogloia* sp*.* | Thwaites ex W.Smith, 1856 |  | X |
| *Mastogloia smithii* | Thwaites ex W.Smith, 1856 | X | X |
| *Melosira varians* | C.Agardh, 1827 | X | X |
| *Navicula* sp1*.* | Bory de Saint-Vincent, 1822 | X | X |
| *Navicula* sp2*.* | Bory de Saint-Vincent, 1822 | X | X |
| *Navicula capitatoradiata* | Germain, 1981 |  | X |
| *Nitzschia* spp*.* | Baer, 1826 | X | X |
| *Cylindrotheca closterium* | (Ehrenberg) Reimann & J.C.Lewin, 1964 | X | X |
| *Pseudo-nitzschia* spp. | (G.R.Hasle) G.R.Hasle, 1993 | X | X |
| *Nitzschia sigmoidea* | (Nitzsch) W.Smith, 1853 |  | X |
| *Odontella* sp*.* | C.Agardh, 1832 | X |  |
| *Paralia sulcata* | (Ehrenberg) Cleve, 1873 | X |  |
| *Pleurosigma* spp*.* | W.Smith, 1852 | X | X |
| *Proboscia alata* | Sundström 1986 | X | X |
| *Pseudosolenia* sp 1*.* | B.G.Sundström, 1986 | X | X |
| *Pseudosolenia* sp 2 | B.G.Sundström, 1986 | X | X |
| *Rhizosolenia imbricata* | Brightwell, 1858 | X | X |
| *Rhizosolenia styliformis* | T.Brightwell, 1858 | X | X |
| *Rhizosolenia setigera* | Brightwell, 1858 | X |  |
| *Skeletonema costatum* | (Greville) Cleve, 1873 | X | X |
| *Stephanodiscus* spp*.* | Ehrenberg, 1845 | X |  |
| *Stephanopyxis* spp*.* | (Ehrenberg) Ehrenberg, 1845 | X |  |
| *Striatella unipunctata* | C.Agardh 1832 | X | X |
| *Surirella minuta* | Turpin 1828 | X | X |
| *Surirella* spp*.* | Turpin 1828 |  | X |
| *Thalassionema* spp*.* | Brébisson in Kützing 1849 | X | X |
| *Thalassionema nitzschioides* | (Grunow) Mereschkowsky, 1902 | X | X |
| *Thalassiosira anguste-lineata* | (A.Schmidt) G.Fryxell & Hasle, 1977 | X | X |
| *Thalassiosira eccentrica* | (Ehrenberg) Cleve, 1904 | X | X |
| *Thalassiosira gravida* | Cleve, 1896 | X | X |
| *Thalassiosira subtilis* | (Ostenfeld) Gran, 1900 | X | X |
| *Triceratium elegans* | (Greville) Grunow |  | X |
| *Triceratium majus* | (E.Grove & G.Sturt) E.Grove & G.Sturt |  | X |
| **Haptophyta** |  |  |  |
| *Emiliania huxleyi* | (Lohmann) W.W.Hay & H.P.Mohler, 1967 | X | X |
| **Chlorophyta** |  |  |  |
| *Eudorina* sp*.* 1 | Ehrenberg, 1832 | X |  |
| *Pandorina* sp*.* 1 | Bory de Saint-Vincent, 1824 | X |  |
| *Gonium* sp. 1 | O.F.Müller, 1773 |  | X |
| **Cyanobacteria** |  |  |  |
| *Chroococcus* spp*.* | Nägeli, 1849 | X | X |
| *Anabaena* sp*.* 1 | Bory de Saint-Vincent ex Bornet & Flahault, 1886 | X |  |
| *Anacystis* spp*.* | Meneghini, 1837 | X |  |
| *Arthrospira* spp*.* | Sitzenberger ex Gomont, 1892 | X |  |
| *Aphanizomenon* spp*.* | A.Morren ex Bornet & Flahault, 1888 | X |  |
| *Lyngbya* spp*.* | C.Agardh ex Gomont, 1892 | X |  |
| *Synechocystis* spp*.* | Sauvageau, 1892 | X |  |
| **Euglenozoa** |  |  |  |
| *Eutreptiella* sp. 1 | A. da Cunha, 1914 | X | X |
| *Euglena* sp. 1 | Ehrenberg, 1830 |  | X |
| **Myzozoa** |  |  |  |
| **Dinophyceae** |  |  |  |
| *Alexandrium* spp*.* | Halim, 1960 | X |  |
| *Alexandrium minutum* | Halim, 1960 | X |  |
| *Alexandrium tamarense* | (Lebour) Balech, 1995 | X |  |
| *Tripos azoricus* | (Cleve) F.Gómez, 2013 | X | X |
| *Tripos macroceros* | (Ehrenberg) F.Gómez, 2013 | X |  |
| *Dinophysis* spp*.* | Ehrenberg, 1839 | X |  |
| *Dinophysis acuminata* | Claparède & Lachmann, 1859 | X |  |
| *Gonyaulax* spp. | K.M.Diesing, 1866 | X |  |
| *Gonyaulax ceratocoroides* | Kofoid, 1910 | X |  |
| *Karenia brevis* | (C.C.Davis) Gert Hansen & Ø.Moestrup, 2000 | X |  |
| *Gyrodinium spirale* | (Bergh) Kofoid & Swezy, 1921 | X |  |
| *Gymnodinium catenatum* | HW Graham 1943 | X | X |
| *Neoceratium lineatum* | (Ehrenberg) Cleve, 1899 | X | X |
| *Noctiluca scintillans* | Suriray, 1836 | X |  |
| *Ornithocercus steinii* | Schütt, 1900 | X |  |
| *Ostreopsis sp.* | J.Schmidt, 1901 | X |  |
| *Oxytoxum scolopax* | Stein, 1883 | X |  |
| *Preperidinium* spp*.* | Mangin, 1913 | X |  |
| *Prorocentrum* sp*.* 1 | Ehrenberg, 1834 | X |  |
| *Prorocentrum micans* | Ehrenberg, 1834 | X |  |
| *Protoperidinium* sp.1 | Bergh, 1882 | X |  |
| *Protoperidinium conicoides* | (Paulsen) Balech, 1973 | X |  |
| *Protoperidinium excentricum* | (Paulsen) Balech, 1974 | X |  |
| *Archaeperidinium minutum* | (Kofoid) Jørgensen, 1912 | X |  |
| *Protoperidinium pyriforme* | (Paulsen) Balech, 1974 | X |  |
| *Protoperidinium thorianum* | (Paulsen) Balech, 1974 | X |  |
| *Scrippsiella trochoidea* | (Stein) Loeblich III, 1976 | X | X |
| *Sinophysis* spp. | Nie & C.Wang, 1944 | X | X |
