# Supplementary material for: Microphytoplankton variations during coral spawning at Los Roques, Southern Caribbean
Source: PeerJ. 2016 Mar 17;4:e1747. doi: 10.7717/peerj.1747 (PMC4806606; doi:10.7717/peerj.1747)
Supplement: Table S2 [file peerj-04-1747-s002.docx]

**Supplementary Table 2.** Total density of the microalgae species that contributed with more than 60% of the dissimilarity between spawning times evaluated in 2007 and 2008 at Los Roques, Venezuela, Southern Caribbean. Values shown represent the sum of the four replicates taken at each reef in cells/mL.

| **DIATOMS** | | | | | | | | |
| --- | --- | --- | --- | --- | --- | --- | --- | --- |
| **Lo** | **Reef** | **Period** | ***T. subtilis*** | | ***C. pelagica*** | | ***R. imbricata*** | |
|  |  |  | **2007** | ***2008*** | ***2007*** | ***2008*** | ***2007*** | ***2008*** |
| NE | GR | Before | 5.421 | 2.0957 | 1.08 | 0.3477 | 3.012 | 0.1028 |
|  |  | During | 2.94 | 0.333 | 0.96 | 0.3342 | 10.957 | 1.2352 |
|  |  | After | 2.27 | 0.754 | 4.067 | 0.5275 | 4.487 | 0.8252 |
|  | MD | Before | 4.65 | 1.9194 | 1.032 | 0.2606 | 13.543 | 0.1162 |
|  |  | During | 6.09 | 0.9794 | 0.982 | 0.309 | 10.127 | 0.4582 |
|  |  | After | 11.87 | 1.6874 | 6.118 | 0.153 | 5.275 | 0.6699 |
| SW | DMS | Before | 17.643 | 1.0839 | 5.842 | 0.4338 | 6.375 | 0.2099 |
|  |  | During | 12.75 | 2.0645 | 5.374 | 0.887 | 6.832 | 0.6821 |
|  |  | After | 7.054 | 1.5575 | 7.153 | 0.1324 | 3.851 | 0.4548 |
|  | CYA | Before | 8.324 | 1.3818 | 5.479 | 0.7968 | 4.521 | 0.3319 |
|  |  | During | 10.15 | 1.5348 | 4.958 | 1.3078 | 4.952 | 0.8904 |
|  |  | After | 5.83 | 0.2321 | 6.025 | 0.613 | 2.571 | 0.4601 |
| **DINOFLAGELLATES** | | | | | | | | |
| **Lo** | **Reef** | **Period** | ***Protoperidinium* species** | | ***S. trochoidea*** | | ***N. lineatum*** | |
|  |  |  | 2007 | 2008 | 2007 | 2008 | 2007 | 2008 |
| NE | GR | Before | 2.237 | 0.4383 | 3.645 | 1.12 | 3.974 | 3.446 |
|  |  | During | 7.942 | 0 | 4.376 | 2.374 | 4.865 | 2.975 |
|  |  | After | 5.2847 | 0 | 4.965 | 4.155 | 7.5432 | 4.9168 |
|  | MD | Before | 6.5574 | 0 | 4.51 | 0 | 8.91 | 0 |
|  |  | During | 6.5974 | 0 | 4.32 | 0 | 8.32 | 0 |
|  |  | After | 18.3574 | 0 | 6.04 | 0 | 9.24 | 0 |
| SW | DMS | Before | 13.4115 | 0 | 6.243 | 0 | 21.875 | 0 |
|  |  | During | 19.6145 | 0 | 3.53 | 0 | 17.32 | 0 |
|  |  | After | 23.5269 | 0 | 4.13 | 0 | 11.85 | 0 |
|  | CYA | Before | 14.5058 | 0 | 3.12 | 0 | 5.32 | 0 |
|  |  | During | 21.5778 | 0 | 1.87 | 0 | 2.43 | 0 |
|  |  | After | 29.3317 | 0 | 0.64 | 0 | 3.65 | 0 |

**Lo:** Locality
