# Supplementary material for: Microphytoplankton variations during coral spawning at Los Roques, Southern Caribbean
Source: PeerJ. 2016 Mar 17;4:e1747. doi: 10.7717/peerj.1747 (PMC4806606; doi:10.7717/peerj.1747)
Supplement: Table S3 — Acropora palmata larvae abundance, measured through specific antibody signal during spawning times evaluated in 2007 and 2008 at Los Roques, Venezuela, Southern Caribbean. Values shown represent the average absorbance of the four replicates taken at each reef. [file peerj-04-1747-s004.docx]

**Supplementary Table 3.** *Acropora palmata* larvae abundance, measured through specific antibody signal during spawning times evaluated in 2007 and 2008 at Los Roques, Venezuela, Southern Caribbean. Values shown represent the average absorbance of the four replicates taken at each reef.

|  | YEAR | | | | | | | | | | | | |
| --- | --- | --- | --- | --- | --- | --- | --- | --- | --- | --- | --- | --- | --- |
|  | 2007 | | | | | |  | | 2008 | | | | |
| **Period** | Before | During | After | Before | During | After | Before | During | | After | Before | During | After |
| Cayo Agua | 0.11 ± 0.07 | 0.07 ± 0.01 | 0.06 ± 0.003 | 0.06 ± 0.004 | 0.06 ± 0.01 | 0.06 ± 0.01 | 0.15 ± 0.09 | 0.13 ± 0.21 | | 0.08 ± 0.02 | 0.05 ± 0.03 | 0.1 ± 0.05 | 0.07 ± 0.01 |
| Dos Mosquises | 0.08 ± 0.03 | 0.07 ± 0.01 | 0.06 ± 0.01 | 0.05 ± 0.003 | 0.08 ± 0.12 | 0.1 ± 0.16 | 0.19 ± 0.1 | 0.08 ± 0.02 | | 0.07 ± 0.01 | 0.07 ± 0. | 0.1 ± 0.04 | 0.07 ± 0. |
| Madrizquí | 0.08 ± 0.03 | 0.07 ± 0.01 | 0.07 ± 0.02 | 0.05 ± 0.004 | 0.06 ± 0.01 | 0.08 ± 0.11 | 0.11 ± 0.09 | 0.07 ± 0.004 | | 0.14 ± 0.21 | 0.04 ± 0.03 | 0.08 ± 0.03 | 0.08 ± 0.03 |
| Gran Roque | 0.08 ± 0.03 | 0.07 ± 0.01 | 0.08 ± 0.03 | 0.05 ± 0.004 | 0.06 ± 0.01 | 0.1 ± 0.15 | 0.15 ± 0.12 | 0.07 ± 0.004 | | 0.08 ± 0.01 | 0.07 ± 0.01 | 0.08 ± 0.02 | 0.08 ± 0.03 |
|  |  |  |  |  |  |  |  |  | |  |  |  |  |
|  |  |  |  |  |  |  |  |  | |  |  |  |  |
| Average | 0.11 | 0.07 | 0.06 | 0.06 | 0.06 | 0.06 | 0.15 | 0.13 | | 0.08 | 0.05 | 0.10 | 0.07 |
|  | 0.08 | 0.07 | 0.06 | 0.05 | 0.08 | 0.10 | 0.19 | 0.08 | | 0.07 | 0.07 | 0.10 | 0.07 |
|  | 0.08 | 0.07 | 0.07 | 0.05 | 0.06 | 0.08 | 0.11 | 0.07 | | 0.14 | 0.04 | 0.08 | 0.08 |
|  | 0.08 | 0.07 | 0.08 | 0.05 | 0.06 | 0.10 | 0.15 | 0.07 | | 0.08 | 0.07 | 0.08 | 0.08 |
|  |  |  |  |  |  |  |  |  | |  |  |  |  |
| SD | 0.067858548 | 0.012579417 | 0.003486192 | 0.003665102 | 0.006811519 | 0.009047279 | 0.085388508 | 0.205272349 | | 0.017507905 | 0.033794268 | 0.054772499 | 0.005769255 |
|  | 0.029467027 | 0.005144603 | 0.012424769 | 0.003361116 | 0.118546443 | 0.157036874 | 0.099667675 | 0.019173077 | | 0.010241623 | 0.004919647 | 0.040160046 | 0.004636223 |
|  | 0.030584338 | 0.011944372 | 0.016281822 | 0.003740689 | 0.005163978 | 0.108874845 | 0.093932315 | 0.003930427 | | 0.20595759 | 0.031972821 | 0.025557888 | 0.027872599 |
|  | 0.032164487 | 0.013488115 | 0.0257982 | 0.003566907 | 0.006371927 | 0.153547183 | 0.117602686 | 0.00421243 | | 0.009397401 | 0.005704015 | 0.023825527 | 0.026511814 |

**SD: Standard Deviation**
